# Genomic Insight into the Role of lncRNAs in Cancer Susceptibility

**DOI:** 10.3390/ijms18061239

**Published:** 2017-06-09

**Authors:** Ping Gao, Gong-Hong Wei

**Affiliations:** Biocenter Oulu, Faculty of Biochemistry and Molecular Medicine, University of Oulu, Oulu 90014, Finland; ping.gao@oulu.fi

**Keywords:** lncRNA, GWAS, genomics, transcription factor, SNP, cancer

## Abstract

With the development of advanced genomic methods, a large amount of long non-coding RNAs (lncRNAs) have been found to be important for cancer initiation and progression. Given that most of the genome-wide association study (GWAS)-identified cancer risk SNPs are located in the noncoding region, the expression and function of lncRNAs are more likely to be affected by the SNPs. The SNPs may affect the expression of lncRNAs directly through disrupting the binding of transcription factors or indirectly by affecting the expression of regulatory factors. Moreover, SNPs may disrupt the interaction between lncRNAs and other RNAs or proteins. Unveiling the relationship of lncRNA, protein-coding genes, transcription factors and miRNAs from the angle of genomics will improve the accuracy of disease prediction and help find new therapeutic targets.

## 1. Introduction

The non-coding region spans approximately 97.2% of the human genome in comparison to the protein coding region [1]. With the development of high-throughput sequencing methods, a large amount of non-coding RNAs including lncRNAs have been found in mammalian cells. In the beginning, most non-coding RNAs were assumed to have no function and treated as the waste of transcripts [2]. However, in recent years, new techniques have been developed to show that non-coding RNAs do have important functions in cells. Non-coding RNAs are connected with epigenetic disturbance. For example, lncRNA *MYCN* opposite strand (*MYCNOS*) can speed up chromatin remodeling via cooperating with CCCTC-binding factor (CTCF). CTCF plays a crucial role in regulating the 3D structure of chromatin. CTCF cooperates with *MYCNOS* to increase the expression of oncogene *MYCN* by facilitating chromatin remodeling [3]. Moreover, the RNA transcribed from regulatory elements also plays roles in moderating the function of transcription factors in regulating target genes. Tethering the RNA transcribed from the transcription factor Yin-Yang 1 (YY1) binding site using the CRISPR/Cas9 system could enhance the binding occupancy of YY1 to its binding site [4]. Many studies also unveiled the crucial role of non-coding RNA including lncRNAs in tumorigenesis and cancer progression.

## 2. Novel lncRNAs Found in Different Types of Cancers

lncRNA is a kind of non-coding RNA, which is longer than 200 bp and does not encode a protein. lncRNAs can be classified into intergenic, intronic, exonic, overlapping and antisense lncRNA based on their genomic location [5]. lncRNAs were recently found to be functionally important in cancer initiation and progression.

### 2.1. lncRNAs Have Been Found in Many Cancer Types

Some lncRNAs have been found to be associated with many types of cancers. Table 1 lists the example of the lncRNAs in different cancer types. For example, by analyzing 40 clinical bladder cancer tissues and four cell lines, small nucleolar RNA host gene 16 (*SNHG16*) was discovered with high expression in bladder, lung and colorectal cancer [6,7,8]. It promotes cell growth through the Wnt signaling pathway or cooperates with *miR-98*. Hypoxia-inducible factor-1α antisense transcript (*HIF1A-AS*) is also a well-established oncogene associated with renal cancer [9,10]. Two lncRNAs named *MALAT1* and *H19* were found to be oncogenes in lung cancer and other cancer types [11,12,13,14,15,16]. Both of them need to interact with microRNAs to execute functions. For example, *miR-138*, which inhibits the expression of *HMGA2*, was targeted by *H19*. Moreover, 568 transcripts were found upregulated and 740 transcripts downregulated in renal cell carcinoma (ccRCC) tissue by analyzing the microarray data from 15 malignant renal tumors and matching normal tissues [17].

### 2.2. lncRNAs in Prostate Cancer

By analyzing the RNA-seq, microarray and qPCR data of tumor and normal tissues, many lncRNAs were found overexpressed in the tumor tissues [25]. Table 2 summarizes the lncRNAs that have been found in prostate cancer. Prostate cancer-associated transcript 3 (*PCA3*) was identified to be specifically expressed in prostate cancer cells [26]. In comparison with serum PSA (prostate specific antigen), *PCA3* is less sensitive, but more specific in detecting prostate cancer. Another lncRNA called solute carrier family 45 member 3 (*SChLAP1*) can promote prostate cancer cell invasion and metastasis and is useful in predicting lethal prostate cancer [27]. Back to 2000, another group found that prostate-specific transcript 1 (*PCGEM1*) was overexpressed in prostate cancer tumors [28]. Recently, one study reported that prostate cancer-associated non-coding RNA 1 (*PRNCR1*) and *PCGEM1* were highly overexpressed in aggressive prostate cancer [29]. These two lncRNAs can interact with AR in androgen-independent cells, suggesting that they play a role in castration-resistance prostate cancer. Controversially, another study found that *PCGEM1,* but not *PRNCR1,* was overexpressed in prostate cancer. There was no interaction between *PCGEM1* and AR [30]. In addition to promoting cell proliferation via AR signaling, some lncRNAs could promote prostate cancer growth by inhibiting DNA repair. For example, BRCA2, a tumor repressor that is important in DNA double break repair, was inhibited by *PCAT1* overexpression [31,32]. In addition, *PCAT1* can promote cell proliferation through cMYC [33].

## 3. Genetic Variants Affect lncRNA Expression

GWASs have facilitated the finding of many genetic variants associated with many different types of traits and diseases, including cancer. The variants can be single nucleotide or several nucleotide differences among individuals. In addition to single nucleotide variation, there are structural variants including more nucleotide and position changes such as insert-deletion variants, block substitutions, inversions and copy number variations [44,45]. Compared to the amount of single nucleotide variants, there are less structural variants, probably due to a lack of the advanced technology to detect them [44,46,47,48,49].

As most of the common genetic variants, single nucleotide polymorphisms (SNPs) are located in the non-coding region; the expression and the function of lncRNAs are more likely to be affected by the SNPs. lncRNAs represent one of the largest classes of non-coding RNAs. Numerous studies have found the association between lncRNAs and many types of human diseases, which raises the important question of whether the SNPs can affect the expression of lncRNA. To test this hypothesis, Kumar et al. investigated the relationship of SNPs and the expression level of SNP-associated lncRNAs [50]. Using genome-wide gene expression and genotype data from 1240 peripheral blood samples and four different types of tissue from 85 individuals, they performed eQTL mapping on 2140 human long intergenic noncoding RNA (lincRNA)-probes and identified tissue-dependent *cis*-eQTLs for lncRNAs. The results showed that 75% of the SNPs affect lncRNA expression (lncRNA *cis*-eQTLs), but not for their neighboring protein-coding genes [50]. Some of these SNPs are associated with disease, suggesting that these SNPs impact health by regulating the expression of lncRNAs. Thus far, many databases about lncRNAs have been built. For example, Gong et al. systematically analyzed the relationship between SNPs and lncRNAs in the genomes of human and mouse [51]. A large amount of SNPs were found to affect the expression of lncRNAs, and 142 of them are GWAS tag SNPs. Additionally, rs2839698 located in *H19* was reported to be associated with bladder cancer. Individuals with genotype TC at rs2839698 have less chance of developing non-muscle-invasive bladder cancer when compared to CC homozygotes [52]. Two SNPs, rs6434568 and rs16834898, within the *PCGEM1* gene are associated with prostate cancer risk in Chinese men, with the C at rs6434568 and A at rs16834898 as risk alleles, respectively [53]. However, how these SNPs contribute to the risk of prostate cancer through *PCGEM1* is still unclear. Unveiling the relationship between SNPs, lncRNAs and disease mechanisms is an important question to investigate.

## 4. Regulatory Mechanisms Underlying Risk SNPs and lncRNAs in Cancer

The location of SNPs is an important factor that needs to be considered when we dissect the mechanisms underlying SNPs and lncRNAs. For example, some SNPs are located at the regulatory elements; some reside in the lncRNAs. Here, we will follow this clue to introduce the different regulatory mechanisms underlying the SNPs and lncRNAs.

### 4.1. SNPs Reside in the lncRNAs

**Figure 1:**
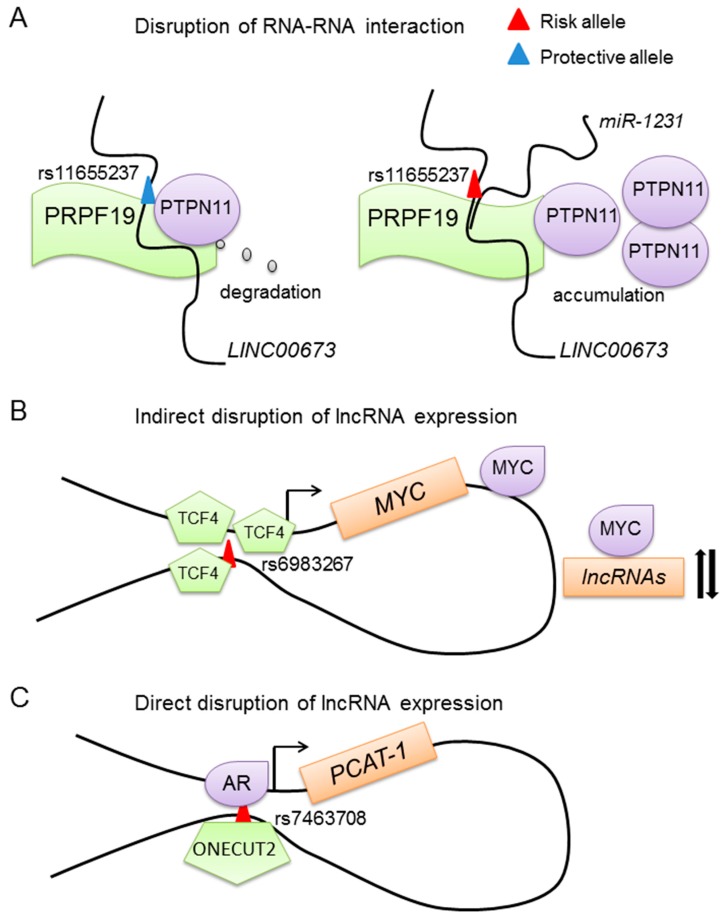
Mechanisms of SNPs in disrupting lncRNA expression and function. (**A**) The risk allele residing in the lncRNA disrupts its interaction with other RNAs or proteins. For example, the risk allele at rs11655237 enhances the interaction between *LINC00673* and *miR-1231*, which blocks the degradation of PTPN11; (**B**) SNPs regulate lncRNA expression by disrupting the expression of other regulatory factors. The risk allele of rs6983267 increases the expression of *MYC*, a transcription factor, which might regulate the expression of many lncRNAs; (**C**) The risk allele directly disrupts lncRNA expression. Shown is the risk allele of SNP rs7463708, inducing the expression of *PCAT-1* through increasing the binding affinity of *ONECUT2* to the SNP-containing region.

The regulatory mechanisms underlying SNPs and lncRNAs might be similar to the regulatory mechanisms related to SNPs and protein-coding genes. One classical mechanism related to the SNPs and protein coding gene is the SNPs residing in the protein coding gene, which may cause amino acid change and disrupt the function of the protein. One example is the SNP rs138213197, a rare variant causing amino acid change of G84E in HOXB13, which frequently appears in DNA samples of 94 families with hereditary prostate cancer [54]. The results indicate that HOXB13 G84E is a heritable variation associated with prostate cancer. The T allele at rs138213197 is associated with a higher risk of prostate cancer [55,56]. The variation might disrupt the binding ability and specificity in HOXB13-mediated protein-protein interactions. Given that HOXB13 is known to interact with AR signaling and plays an important role in prostate development, the HOXB13 variation may also disrupt the AR pathway and promote prostate cancer initiation and progression. Similar mechanisms may also occur in lncRNAs. For example, the lung cancer risk SNP rs114020893 at 1p31.1 residing in lncRNA *NEXN-AS1* might change its secondary structure [57]. Two SNPs named rs2288947 and rs8105637 that reside in tissue differentiation-inducing non-protein coding RNA (*TINCR*) are associated with colorectal cancer. Inhibiting the expression of *TINCR* results in the activation of EpCAM cleavage, which will promote the proliferation and metastasis of colorectal cancer. The protective or risk allele of these two SNPs might affect the EpCAM cleavage through *TINCR* [58]. One similar example has been found in breast cancer. Variant SNPs located in or near lncRNA *MIR2052HG* are associated with breast cancer [59]. One example found in prostate cancer is the multiple type of cancer risk-related lncRNA HOX transcript antisense RNA (*HOTAIR*). There are two *HOTAIR* polymorphisms named rs12826786 and rs1899663. Taheri et al. analyzed the association between those two SNPs and the risk of prostate cancer and benign prostate hyperplasia (BPH) in a population of 128 Iranian prostate cancer patients, 143 BPH patients and 250 normal male controls [60]. They found that the rs1899663 T allele was associated with BPH risk and the rs12826786 T allele was associated with both BPH and prostate cancer susceptibility. Even though the mechanisms underlying these two SNPs and *HOTAIR* are still unclear, we might find some clues from other non-coding RNAs such as the pancreatic cancer-associated long intergenic noncoding RNA lincRNA *LINC00673* [61]. As shown in Figure 1A, *LINC00673* is a tumor suppressor and can increase the interaction between PTPN11 and an E3 ubiquitin ligase PRPF19. PRPF19 is important in promoting the degradation of the protein PTPN11. However, the risk variant G at rs11655237 in exon 4 of *LINC00673* increases the binding of *miR-1231* to *LINC00673*, which blocks the normal function of this lincRNA and causes an accumulation of PTPN11. Thus, the SNPs that reside in the lncRNAs might also disturb the interaction between the lncRNAs and other RNAs, which in turn affects protein expression. Together, the SNPs located in the lncRNAs might affect the secondary structure of lncRNA and subsequently disrupt the interaction between the lncRNA and other RNAs or proteins.

### 4.2. SNPs Reside Far Away from lncRNAs

As we know, transcription factors play important roles in gene regulation. Some variants such as rs6983267 have been found to increase disease risk by affecting the expression of transcription factors (Figure 1B). It regulates *MYC* expression through disrupting the binding affinity of TCF4 to the SNP containing region [62,63]. This GWAS finding has been verified in a mouse model. Mice with knockout of the rs6983267-containing DNA fragment have slightly reduced transcription of *Myc* in the intestinal crypts and are more resistant to intestinal tumorigenesis than wild-type mice [64]. Moreover, MYC is a transcription factor and plays important roles in tumorigenesis. Aberrant expression of *MYC* might disturb the RNA levels of many genes including lncRNAs.

Another study suggests that the binding of miRNA to the 3′UTR of genes is affected by the genetic variants residing in the genes [65]. They found twenty-two SNPs that are associated with the risk of prostate cancer disrupt miRNA binding (miRSNP). For example, *miR-3162-5p* prefers binding to the T allele of *KLK3* rs1058205; *miR-370* has stronger affinity for the *VAMP8* rs1010 miRSNP A-allele. Based on this finding, we can assume that the SNPs residing in regulatory elements of lncRNAs might also affect the binding of miRNA to lncRNAs. Thus, this represents another mechanism of how SNPs regulate the transcription of lncRNAs.

### 4.3. SNPs Affect lncRNA Expression through Disrupting DNA-Binding of Transcription Factors

Regulatory regions reside in the non-coding region playing crucial roles in gene regulation [66,67]. SNPs residing in the regulatory regions of the lncRNAs can disrupt the binding of transcription factors to the SNP containing region. As shown in Figure 1C, rs7463708 is located in an enhancer region, which is 78 kb downstream of the *PCAT-1* transcription start site (TSS). Through dissecting the mechanisms underlying rs7463708 and *PCAT-1*, He and colleagues found that the risk T allele at rs7463708 enhances the binding of *ONECUT2* to the SNP-containing region. *ONECUT2* has an interaction with AR, and knockdown of *ONECUT2* inhibits the expression of an androgen-induced lncRNA gene *PCAT-1*. Moreover, there is long-rang chromatin interaction between the SNP-containing region and *PCAT-1*. These results suggest that the risk allele of rs7463708 increases the expression of *PCAT-1* through increasing the binding of *ONECUT2* [68].

Similar to this finding, our previous studies found that the prostate cancer risk-associated T allele of SNP rs339331 increases the expression of oncogene *RFX6* by disrupting the binding of transcription factor HOXB13 [69]. Moreover, we recently revealed more examples of SNPs in disrupting the DNA-binding motifs not only of single transcription factor, but also transcription factor–transcription factor complexes [70]. The difference between those findings is the target gene; one is a protein-coding gene, and the other is lncRNA. Taken together, the complicated relationship between lncRNA, the protein coding gene, transcription factors and miRNA suggests that the genetic variants might regulate gene expression and tumorigenesis through different mechanisms.

## 5. Clinical Use of GWAS and lncRNA Findings for Cancer Risk Prediction and Future Remarks

GWAS is a powerful approach to identify disease-associated genetic variants by analyzing a large number of cases and controls, leading to the identification of the majority of the SNPs located at the non-coding region. Therefore, the application of GWAS for studying the association of ncRNAs with diseases should be more promising than just applying GWAS to protein coding genes. However, one major disadvantage would be that the majority of ncRNAs including lncRNAs have an unknown function, and their biological roles are rather difficult to predict in comparison with protein-coding genes. Moreover, one of the general challenges in GWAS is how to translate those findings to clinical diagnosis and treatment [71]. To overcome these disadvantages, we shall initiate post-GWAS studies and deeply dissect the regulatory mechanisms underlying those risk loci [72]. In doing so, it will help us to identify novel biomarkers, improve disease prediction and eventually pinpoint therapeutic targets [73].

Through dissecting the mechanisms underlying GWAS, SNPs and lncRNAs can help us find more causal genes and fully understand the function of those risk SNPs during oncogenesis. In the clinical setting, lncRNAs can be used as biomarkers to check the tumor stage. For example, the urinary biomarker *PCA3* (*DD3*) as described above has been used to diagnose prostate cancer [26]. *PCA3* as a biomarker is more specific than PSA and causes less discomfort compared to a needle biopsy. In bladder cancer patients, high levels of small nucleolar RNA host gene 16 (*SNHG16*) indicate a high bladder cancer lethality. The risk for recurrence in bladder cancer can also be predicted by checking the fraction of cells expressing H19 from biopsies [74]. Although using lncRNAs as biomarkers works well, there are still limitations since the genetic background of individuals is quite different. When we combine our GWAS findings and the lncRNAs together to predict the risk of the disease, our prediction and therapeutic targets might be more accurate. In the future, more genome-wide analyses related to lncRNAs should be performed.

Developing methods to inhibit overexpressed oncogenic lncRNAs is a potential way to treat cancer. Small interfering RNAs (siRNAs) offer a means to target lncRNA [75,76], but it is difficult to design an efficient one since the size of the lncRNA is large [77]. Recently, the modified genome editing tool CRISPR/Cas9 was applied to target the RNA. Nelles et al. successfully applied RNA-targeting Cas9 (RCas9) to track and cleave RNA in liver cells [78]. This advanced technology provides a new strategy to directly modify RNA levels in vivo and is likely to be adapted to degrade oncogenic lncRNAs for cancer therapy. Since lncRNAs might function as enhancer RNAs, resulting in big variation of many genes’ expression, developing tools to target lncRNAs might be more efficient than targeting protein-coding genes.

## Figures and Tables

**Table 1:** List of lncRNAs in cancer.

lncRNA Name	Cancer Type	Potential Mechanism	Reference
*AFAP1-AS1*	Gastric Cancer	Via the PTEN/p-AKT pathway	[18]
*XIST*	Non-Small Cell Lung Cancer	XIST and *miR-186-5*p are likely in the same RNA-induced silencing complex	[19]
*lncRNA CHRF*	Colorectal Cancer	Inhibits *miR-489* expression	[20]
*SNHG16*	Bladder Lung Colorectal	Wnt pathway, binding *miR-98* with E2F5	[6,7,8]
*HIF1A-AS*	Renal Cancer	No report	[9,10]
*MALAT1*	Lung Gastric Osteosarcoma Tongue	Interacts with *miR-124*, *miR-142-3p, miR-129-5p* and *miR-1297*	[11,12,13,14]
*H19*	Lung Colon	Upregulates the expression of HMGA1 by sponging *miR-138*	[15,16]
*TP73-AS1*	Hepatocellular Carcinoma	Inversely correlated with *miR-200a*	[21]
*TUG1*	Endometrial	Inhibiting *miR-299* and *miR-34a-5p*	[22]
*XIST*	Osteosarcoma	Directly binds to *miR-320b* and repressed *miR-320b* expression	[23]
*CCAT1*	Oral Squamous Cell Carcinomas	Through *miR155-5p* and let7b-5p	[24]

**Table 2:** Incomplete list of the prostate cancer-associated lncRNAs.

lncRNA Name	Cancer Type	Potential Mechanism	Reference
Onco-lncRNAs: overexpression in cancer			
*CDKN2B-AS1 (ANRIL*, *p15AS)*	Prostate, others	Epigenetic silencing of the locus by interaction with CBX7 and PRC2	[34,35]
*PCA3/DD3*	Prostate	Modulating AR signaling	[36]
*PCAT-1*	Prostate	Inhibits BRCA2 and activates MYC, silencing gene through PRC2	[32,33]
*PCAT6*	Prostate, others	Oncogenic phenotypic effects, molecular mechanisms are unknown	[37]
*PCAT7*	Prostate, others	Oncogenic phenotypic effects, but molecular mechanisms are unknown	[37]
*PVT1*	Prostate, others	Oncogenic phenotypic effects, molecular mechanisms are unknown	[37]
*PCGEM1*	Prostate	Inhibits apoptosis; promotes cell proliferation	[30]
*MALAT1*	Prostate, others	Alternative splicing of pre-mRNAs	[38]
*HOTAIR*	Prostate, others	Binds and stabilizes AR	[38]
*PlncRNA-1*	Prostate, others	Inhibits AR-targeting microRNAs	[39]
*CTBP1-AS*	Prostate	Androgen-responsive gene	[40]
*SCHLAP1 (PCAT11)*	Prostate	Interacts with the SWIF/SNIF complex	[27]
Tumor suppressor-lncRNAs: reduced expression in cancer			
*PTENP1*	Prostate, others	Binds anti-PTEN miRNA	[38]
*GAS5*	Prostate	Prevents glucocorticoid receptor-induced gene expression	[41,42]
*MEG3*	Prostate, others	Downregulates MDM2 and promotes p53 accumulation	[43]
